# Stakeholder Perceptions of Threatened Species and Their Management on Urban Beaches

**DOI:** 10.3390/ani3041002

**Published:** 2013-10-24

**Authors:** Grainne S. Maguire, James M. Rimmer, Michael A. Weston

**Affiliations:** 1 BirdLife Australia, Suite 2-05, The Green Building, 60 Leicester Street, Carlton, VIC 3053, Australia; 2 Barwon Coast, Ewing Blyth Drive, Barwon Heads, VIC 3227, Australia; E-Mail: james@barwoncoast.com.au; 3 Centre for Integrative Ecology, Faculty of Science, Engineering and the Built Environment, School of Life and Environmental Sciences, Deakin University, 221 Burwood Highway, Burwood, VIC 3125, Australia; E-Mail: mweston@deakin.edu.au

**Keywords:** Hooded Plover, regulation, education, recreationists, sandy shores, wildlife, dogs

## Abstract

**Simple Summary:**

Coastal urbanisation brings humans into contact with beach-dwelling wildlife. Where wildlife are disturbance prone, active management is required to promote coexistence between beach-goers and endangered wildlife. Coexistence relies on people adopting wildlife-sensitive behaviours. This study examines factors, which influence people’s awareness and perceptions of threatened species management in southern Australia, using Hooded Plover *Thinornis rubricollis* management as a model. The inconvenience experienced by beach goers in regard to plover management was low. Awareness and support for plover conservation were high. Frequency of beach use, whether a person was a dog walker, and awareness of the species and its plight, influenced perceptions.

**Abstract:**

We surveyed 579 recreationists regarding management of the threatened, beach-dwelling Hooded Plover *Thinornis rubricollis*. We postulated that: (1) lower awareness of the species and higher ‘inconvenience’ of management would engender less favourable perceptions of conservation and management; and (2) that frequency of beach use and dog ownership may mediate perceptions and levels of awareness and inconvenience. Overall, inconvenience was low while awareness and support for plover conservation were high. Education and awareness strategies were considered less effective than regulations; exclusion and regulations were considered less desirable than on-ground protective measures. Awareness, frequency of beach use and dog walking did not influence the perceived effectiveness of different managements. More frequent beach users had greater awareness of the species and their plight but reported greater inconvenience associated with management. Respondents with high awareness rated the severity of human-related threats higher; low awareness was associated with more inconvenience associated with on-ground protection, and exclusion and regulations. Dog walkers reported more inconvenience associated with exclusions and regulations than non-dog walkers. Dog walkers who used the beach infrequently rated threats significantly higher than frequent beach users. Conservation and education strategies could usefully be tailored to beach users’ level of use and pet ownership.

## 1. Introduction

Sandy shores around the world are becoming increasingly urbanized [1]. Human access to these shores is already high and increasing [2]. Urban areas near the coast often constitute a narrow strip of intense development, which enables easy human access to the shoreline [3]. In Australia, many beaches are close to urban centres (such as cities, satellite or holiday towns). Urbanisation is strongly biased towards the coast with 85% and 25% of the Australian population living within 50 and 3 km, respectively, of the coast [3]. Beaches not directly associated with urban development often host recreationists from urban centres [4], and this can be considered a ‘halo effect’ of urbanization [5]. Coastal urbanisation influences ecological processes (for example scavenging guilds; [6]), and in particular results in high usage of beaches by humans, who can disrupt wildlife life history through disturbance and trampling [7,8,9]. Beaches host a range of fauna, including rich avifaunas [10]. A conservation management challenge exists where threatened species live on beaches, especially where exclusion of people is not feasible or desirable [9]. Under such circumstances, understanding human perceptions mediating management effectiveness is pivotal if threatened species are to persist [11].

Social support plays a critical role in threatened species recovery efforts [12,13]. Perceptions of the public about threatened species and their management are important to foster social and political support for conservation programs [14]. An understanding of public perceptions can be fed into behaviour change strategies to promote coexistence between threatened species and humans [15,16,17,18]. In relation to wildlife management, stakeholder perceptions have been documented mostly for pest management (especially the use of lethal population control [19,20,21]), for large North American carnivores such as bears and wolves (often in relation to reintroductions [17,22,23]), and rarely for threatened species [24,25], especially threatened birds (but see [26,27]). Where people constitute a widespread and major threatening process, perceptions of the public are particularly important, and management efforts rely on high and generally voluntary compliance [11]. One such case is the Hooded Plover *Thinornis rubricollis* of south-eastern Australian beaches, where recreationists inadvertently cause disturbance and crush eggs and chicks. A range of effective management techniques, such as ‘temporary beach closures’ and artificial chick shelters, ultimately rely on cooperation from the public by seeking behavioural adjustments e.g., avoidance of small sections of beach or leashing of dogs [8,28,29]. Few studies [30] consider people’s attitudes towards management of threatened species.

Many factors contribute to perceptions of threatened species management programs [11,24]. We hypothesise that personal and demographic factors are prominent influences on perceptions of threats and management among beach goers [15,24]. Prominent personal factors, which may influence perceptions, are inconvenience and awareness [31]. The ‘inconvenience principle’ suggests that people are more likely to acknowledge threats and be sympathetic towards management where compliance is convenient, *i.e.*, requires little change to pre-existing behaviours [31]. Secondly, we predict that the degree of awareness of the species will alter perceptions of threats and management actions, and that awareness will mediate any relationship between inconvenience and perceptions of threats and managements [24]. 

Compliance with management on beaches can vary substantially between demographic groups [9,32,33]. Two demographic factors likely to interact with inconvenience and awareness are the frequency of beach usage and pet ownership. More frequent users will encounter managements more often and compliance will be sought more frequently (*i.e.*, inconvenience will be high). Alternatively, awareness among frequent beach users may also be high and the potential for stewardship exists. Additionally, there may be demographic groups using the beaches more or less frequently, for example, local recreationists and holiday-makers may use beaches at different frequencies [4,34]. Secondly, dog-walkers highly value off-leash exercise for their dogs, and on Australian beaches normally do not comply with leashing regulations [33]. Thus, we hypothesise that Hooded Plover managements are less convenient for dog-walkers over non-dog walkers, and that this may be evident in their perceptions of inconvenience and management efforts. 

We test these ideas by firstly, characterising inconvenience associated with management and pre-existing awareness of the focal species and relating these to perceptions of management and threats, and secondly characterising user demographics according to frequency of beach use and whether a person is a dog-walker, and exploring how these influence perceptions and levels of awareness. The main study area used in this study constitutes highly urbanised beaches, within “day-trip” distance from the city of Melbourne; we thus regard these beaches as urban.

## 2. Methods

We surveyed 684 people (18 years and older) with access to Victorian beaches, September 2009 to April 2010, using three sampling techniques (see http://www.birdlife.org.au/projects/beach-nesting-birds/research): (1) distributing 290 printed questionnaires on plover beaches, (2) letterbox drops (100) adjacent to plover beaches at Anglesea (38°25'39.82"S, 144°10'42.51"E), Ocean Grove (38°16'15.96"S, 144°32'46.50"E), Point Lonsdale (38°17'5.72"S, 144°36'51.20"E), Queenscliff (38°16'2.68"S, 144°39'41.13"E) and Barwon Heads (38°16'53.78"S, 144°29'31.26"E), and (3) advertising an online questionnaire to beach users [35]. Reply paid envelopes were provided as required and respondents remained anonymous. 

The questionnaire consisted of 23 questions regarding respondent demographics, including beach use and dog ownership, awareness of Hooded Plovers, and perceptions of threats and management. Respondents ranked how seriously they perceived 14 threats to Hooded Plovers using a Likert scale (1 “not very serious” to 5 “very serious”). Eight on-ground protective strategies (managements), five regulations and ten community education strategies were then listed and respondents rated how effective they thought these would be for improving plover conservation (1 “not effective” to 5 “very effective”). A subset of these conservation strategies (15 items) was rated by respondents on a scale of 1 (“I don’t like it/it will inconvenience me greatly”) to 5 (“I like it/it is no inconvenience to me”). Respondents also rated five items regarding their perception of the importance of conserving Hooded Plovers.

### 2.1. Sample

Of the 390 surveys distributed, 102 (26.2%) were returned and 582 online surveys were submitted. We excluded 20 online surveys because of incompleteness and 85 surveys because respondents participated in BirdLife Australia’s beach-nesting birds project, and so did not represent the general public. Thus, 579 surveys were available for analysis (online and mailed surveys were amalgamated for analysis because they did not differ in terms of the sex [χ^2^ = 0.688, df = 1, *p* = 0.407] or age of respondents [χ^2^ = 9.033, df = 7, *p* = 0.250]). Females represented 52.2% of respondents (n = 579), similar to 52.5% of coastal residents (outside Melbourne) who are female [36]. Respondents were aged 55–64 (24.4% [15.6%]), 45–54 (19.3% [18.8%]), 35–44 (16.4% [17.7%]), 25–34 (16.4% [14.5%]), 65–74 (13.0%) [11.5%], 18–24 (7.1% [10.4%]), 75–84 (3.1% [8.5%]) and 85+ (0.2% [3.1%]). Figures in square brackets refer to census data for coastal residents outside Melbourne [36] and suggest no substantial demographic bias of respondents. 

Of 576 respondents, 44.6% were residents of Melbourne, 43.5% were from coastal Victoria, 9.5% from regional ‘inland’ Victoria, 1.7% were from another state within Australia and 0.7% from overseas. Many respondents (34.8%) used the beach several times per year; 15.6% and 15.2% used the beach once or two to three times per month, respectively; 13.8% and 10.7%, one to two and three to five times per week, respectively; and 7.3% used the beach daily. Only 2.6% of respondents visited the beach once per year. Overall, 78.5% of respondents visited known Hooded Plover locations (n = 578; visiting 1.71 ± 0.07 Hooded Plover beaches; 0–14). Walking and swimming were the most common beach activity (88.4% and 64.6% of 577 respondents, respectively). 

### 2.2. Data Analysis

Scaled data were analysed using *SPSS* (v. 11.5, *SPSS* Inc., Chicago, IL, USA). Sample sizes vary because not all questions were answered. Means ± one standard error are presented throughout. Factor analysis (principal components analysis [PCA] with varimax rotation) was used to identify groupings of questions (factors) to facilitate interpretation of the data, that is, PCA effectively identified themes used by participants when they answered questions. Items were selected for a factor if they had a component value of greater than 0.5 and factors were considered reliable if Cronbach’s α > 0.5. Repeated measures analysis of variance (ANOVA) with an α level of 0.05 were conducted to compare factor scores (*i.e.*, the average of the item scores within a factor). 

## 3. Results

### 3.1. Characterising Inconvenience

**Figure 1:**
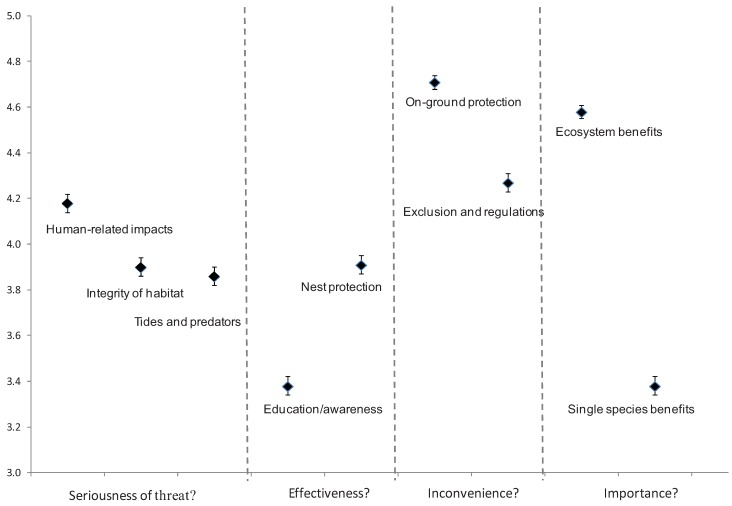
Mean scores (± one standard error) for each factor (theme) as revealed by factor analysis for four separate questions: (1) how serious do you think each threat is, (2) how effective do you think these conservation strategies would be at helping the birds, (3) to what degree would these conservation strategies impact you, and (4) do you think the Hooded Plover is important?

Respondents reported little inconvenience to 15 conservation actions (Table 1). Control of introduced pests, and use of wooden shelters, temporary notices and signs around breeding sites were favoured. Permanently fencing off dunes or temporarily closing an access path were considered slightly less convenient. Factor analysis revealed two reliable factors (themes) that explained the variance in how respondents were personally impacted by conservation actions (Table 2; Figure 1). Mean scores differed between factors (within-subject factor, F_1,520_ = 218.129, *p* < 0.001) with exclusion and regulations considered less favourable than on-ground protection measures (except for prohibition of dune boarding).

**Table 1:** Respondent ratings of the degree of inconvenience perceived in relation to 15 conservation actions rated on a scale from 1 (“I don’t like it/it will inconvenience me greatly”) to 5 (“I like it/it is no inconvenience to me”). Conservation actions are categorised as OG (on-ground actions), RG (regulations) and ED (education/awareness raising actions).

Type of CA	Conservation Actions (CA)	95% confidence intervals	N
OG	1. Control of introduced pests such as foxes and feral cats.	4.79–4.85	573
OG	2. Wooden chick shelters placed along the beach as refuges for chicks to run and hide in when disturbed.	4.75–4.81	571
OG	3. Temporary notices at the beach (alerting me to nests/chicks on the beach).	4.74–4.80	571
OG	4. Signs around the nesting site (these are placed 50–100 m apart around the nesting area, on the beach above the high-tide mark, to delineate the area you are not allowed to use).	4.73–4.79	571
RG	5. Enforcement of regulations.	4.70–4.77	571
RG	6. Dune boarding prohibited.	4.69–4.76	572
ED	7. Interpretive signs at the beach.	4.68–4.74	571
OG	8. Temporarily fencing off the nesting area (this is usually a 50–100 m section of beach that you are restricted from using but can walk past along the water’s edge).	4.64–4.71	571
ED	9. Ranger patrols (rangers give warnings and educational messages for all first offenders).	4.61–4.69	568
RG	10. Horses prohibited.	4.52–4.60	572
ED	11. Face-to-face education.	4.48–4.56	570
RG	12. Dogs allowed, but on leashes only during the breeding season.	4.28–4.38	572
RG	13. Dogs prohibited during the breeding season.	4.26–4.37	571
OG	14. Closure of an access path that enters the beach close to a nesting area for the 63 days it takes to nest and raise a chick.	4.10–4.20	571
OG	15. Permanently fencing off the dunes.	4.02–4.13	570

**Table 2:** Summary of factor analysis results for four questions regarding threats and conservation management for the Hooded Plover. The factors (themes) described by each analysis, the questionnaire items these encompass and summary statistics are provided. Full item descriptions can be cross-referenced from the relevant tables as indicated. Mean factor scores are provided and these are plotted in Figure 1.

Question	Factor (Cronbach’s α; Percentage of variance explained)	Items included in factor (table reference for item descriptions)	Mean factor score (± s.e.)
How serious you think each threat is?	Human-related impacts (0.879; 41.381)	1, 3, 4, 6, 7, 9, 12 (Table 3)	4.18 ± 0.04
	Integrity of habitat (0.746, 11.967)	5, 8, 10, 11, 13 (Table 3)	3.90 ± 0.04
	Tides and predators ^+^ (0.385, 7.630)	2, 14 (Table 3)	3.86 ± 0.04
How effective do you think these conservation strategies would be at helping the birds?	Education/Awareness (0.909, 33.429)	12, 14, 16, 17, 19, 20, 21, 22, 23 (Table 4)	3.38 ± 0.04
	Nest protection (0.819, 10.592)	6, 9, 13, 15 (Table 4)	3.91 ± 0.04
	Regulations (0.780, 7.781)	1, 3, 4, 5, 11 (Table 4)	4.25 ± 0.03
	Exclusion (0.519, 4.895)	8, 10 (Table 4)	4.02 ± 0.04
To what degree would these conservation strategies impact you?	On-ground protection (0.938, 53.132)	1, 2, 3, 4, 5, 6, 7, 8, 9, 11 (Table 1)	4.71 ± 0.03
	Exclusion and regulations (0.741, 9.472)	10, 13, 14, 15 (Table 1)	4.27 ± 0.04
Do you think saving the Hooded Plover is important?	Ecosystem benefits (0.844, 50.661)	1, 2, 3 (Table 5)	4.58 ± 0.03
	Single species benefits (0.633, 25.152)	4, 5 (Table 5)	3.38 ± 0.04

^+^ refers to unreliable factors.

### 3.2. Characterising Awareness

A respondent was ‘aware’ if they had heard of the Hooded Plover and were not confusing it with the Masked Lapwing *Vanellus miles* (an aggressive, super abundant species, colloquially referred to as ‘plover’, and commonly confused with it [37]). A total of 93.7% of 579 respondents had heard of the Hooded Plover, of which 68.2% (538) reported seeing the species and 77.3% (539) knew the difference between Hooded Plover and Masked Lapwing. 

### 3.3. Characterising Perceptions of Threats and Management

Respondents rated the seriousness of 14 known threats to plover eggs and chicks. Disturbance to incubating birds by people and dogs, as well as depredation of eggs and chicks by foxes, ravens and raptors, were considered the greatest threats. High tides and storms, and beach pollution were considered least problematic (Table 3). Factor analysis revealed two reliable factors (Table 2; Figure 1). Mean scores differed between factors (within-subject factor, F_1,494_ = 110.528, *p* < 0.001) with human-related threats being considered more impactful than threats to the integrity of habitat.

Respondents rated the effectiveness of 23 conservation actions at improving Hooded Plover breeding success where one was “not very effective”. Enforcement of regulations was considered most effective and control of introduced pests and prohibition of dune boarding were regarded as highly effective strategies. Educational strategies such as social networks, email updates and website information were considered least effective (Table 4). Factor analysis revealed four reliable factors (Table 2; Figure 1) whose means differed (within-subject factor, F_1,494_ = 136.329, *p* < 0.001) with education and awareness strategies being considered least effective and regulations most effective.

**Table 3:** Respondent ratings of the seriousness of 14 known threats to the Hooded Plover (descending order of importance, where 5 is “serious” and 1 “not serious”). Ninety-five percent confidence intervals are presented.

Threats	95% confidence intervals	N
1. People or dogs disturbing adults from sitting on eggs.	4.51–4.57	572
2. Predators, such as foxes, ravens and hawks, eating eggs and chicks.	4.44–4.51	575
3. Dogs chasing the birds and chicks on the beach.	4.41–4.49	575
4. People or dogs disturbing chicks from feeding.	4.38–4.46	567
5. Loss of habitat.	4.31–4.40	572
6. Dogs crushing eggs when running on the beach/dunes.	4.12–4.21	569
7. People stepping on eggs when walking on the upper beach.	4.03–4.12	572
8. People stepping on eggs when walking in the dunes.	3.90–4.00	574
9. People sitting or sunbaking close to the nest.	3.87–3.95	574
10. Vehicles on beaches.	3.83–3.93	569
11. Erosion of the dunes.	3.61–3.70	572
12. Horses on beaches.	3.55–3.65	570
13. Beach pollution.	3.38–3.48	573
14. Natural threats, such as high tides and storms.	3.17–3.27	573

**Table 4:** Respondent ratings of the perceived effectiveness of 23 conservation actions at improving plover reproductive success, on a scale from 1 (“not very effective”) to 5 (“very effective”). Conservation actions are categorised as OG (on-ground actions), RG (regulations) and ED (education/awareness raising actions).

Type of CA	Conservation Action (CA)	95% confidence intervals	N
RG	1. Enforcement of regulations.	4.58–4.65	570
OG	2. Control of introduced pests such as foxes and feral cats.	4.34–4.41	572
RG	3. Dune boarding prohibited.	4.31–4.40	570
RG	4. Dogs prohibited during the breeding season.	4.18–4.27	569
ED	5. Ranger patrols.	4.11–4.19	568
OG	6. Temporarily fencing off the nesting area (this is usually a 50–100 m section of beach that you are restricted from using but can walk past along the water’s edge).	4.06–4.15	568
OG	7. Wooden chick shelters placed along the beach as refuges for chicks to run and hide in when disturbed.	4.02–4.11	567
OG	8. Closure of an access path that enters the beach close to a nesting area for the 63 days it takes to nest and raise a chick.	3.99–4.08	569
OG	9. Signs around the nesting site (these are placed 50–100 m apart around the nesting area, on the beach above the high-tide mark, to delineate the area you are not allowed to use).	3.96–4.05	571
OG	10. Permanently fencing off the dunes.	3.94–4.04	569
RG	11. Horses prohibited.	3.88–3.98	570
ED	12. Face-to-face education.	3.87–3.96	570
OG	13. Temporary notices at the beach (info. about current nests/chicks on the beach).	3.74–3.82	572
ED	14. Awareness raising events such as coastal beach walks or ‘dogs breakfasts’ to learn about the birds.	3.68–3.76	569
ED	15. Interpretive signs at the beach.	3.66–3.76	569
ED	16. Newspaper/magazine articles.	3.44–3.53	570
ED	17. Brochures about the birds.	3.42–3.51	569
RG	18. Dogs allowed, but on leashes only during the breeding season.	3.38–3.49	571
ED	19. Local radio.	3.33–3.42	569
ED	20. Email updates to alert you to nests in your local area.	3.14–3.24	566
ED	21. Free merchandise such as calendars/bookmarks/stickers to promote the birds.	3.06–3.15	563
ED	22. Website information such as the BirdLife Australia webpage.	3.04–3.14	566
ED	23. Facebook, Myspace or Twitter.	2.89–2.99	561

Respondents rated agreement with five statements about whether it was worth conserving Hooded Plovers (Table 5) and factor analysis revealed two reliable factors, one related to ecosystem benefits of conservation and the second related to the single species benefits of conservation (Table 2; Figure 1). Mean scores differed between factors (within-subject factor, F_1,510_ = 2444.101, *p* < 0.001) with higher support for the importance of the species conservation in an ecosystem context than for the importance of a single species.

**Table 5:** Support for conservation statements rated on a scale from 1 (“strongly disagree”) to 5 (“strongly agree”). Ninety-five percent confidence intervals are presented.

Statement	95% confidence intervals	N
1. It is a unique Australian animal and is important to coastal biodiversity.	4.63–4.70	519
2. People need to reduce their “ecological footprint” and learn to modify their behaviour.	4.54–4.61	518
3. Signing and fencing are relatively cheap and effective managements.	4.44–4.52	516
4. This is an important Australian species under threat.	3.01–3.13	519
5. The onus should not be on the bird to lay its eggs in safer places.	3.66–3.74	516

### 3.4. Characterising Frequency of Beach Use and Dog Walking

To balance sample sizes, data on the frequency of beach use were pooled into three categories: yearly (once a year, several times/year; n = 216), monthly (once/month, two to three times/month; n = 178) and weekly rates of use (one to two and three to five times/week, daily; n = 184). Dog owners represented 38.9% of respondents. Regionally, numbers of dog owners and non-dog owners completing the survey were similar with the exception of Melbourne where non-dog owners accounted for 68.1% of the sample. A total of 73.4% of dog owners indicated that they walked their dog on the beach, of which 78.4% (n = 167) walked their dog off the lead. Overall, 22.6% of all respondents indicated that they walked dogs off leads at the beach. 

### 3.5. Inconvenience, Awareness and Perceptions of Threats and Management

Awareness of Hooded Plovers significantly influenced respondents rating of the severity of threats to Hooded Plovers; people with poor to no awareness of the birds scored human-related impacts lower than those who were aware of the birds (Table 6; 3.88 ± 0.07 *vs.* 4.30 ± 0.04, respectively). Respondents with greater awareness rated exclusions, such as permanent fencing, as less effective than respondents with no or poor awareness (Table 6; 3.97 ± 0.05 *vs.* 4.12 ± 0.07, respectively). Respondents with poor or no awareness felt slightly more inconvenienced than those with awareness of on-ground protection (Table 6; 4.55 ± 0.07 *vs.* 4.77 ± 0.03) and exclusion and regulations (Table 6; 4.07 ± 0.09 *vs.* 4.34 ± 0.04).

**Table 6:** ANOVA output on mean PCA scores for each identified reliable factor. ‘Aware HP’ refers to whether respondents were aware of Hooded Plovers and were not confusing this with awareness of the Masked Lapwing (0 = No, 1 = Yes), Convenience factor 1 (on-ground managements) and Convenience Factor 2 (regulations and exclusion) are mean factor scores derived from a direct question to respondents about how inconvenienced they would be by conservation actions. Significant results are denoted as * = *p* < 0.10 and ** = *p* < 0.05.

Question	Factor	Convenience factor 1: on-ground works	Convenience factor 2: regulations and exclusion	Aware HP
Threats	Human related-impacts	F = 5.044,	F = 23.947,	F = 20.837,
	(r^2^ = 0.178)	*p* = 0.025 ** (+)	*p* < 0.001 ** (+)	*p* < 0.001 **
	Integrity of habitat	F = 3.512,	F = 11.924,	F = 0.821,
	(r^2^ = 0.072)	*p* = 0.062 * (+)	*p* = 0.001 ** (+)	*p* = 0.365
Effectiveness actions	Education/Awareness	F = 20.552,	F = 0.555,	F = 0.454,
	(r^2^ = 0.071)	*p* < 0.001 ** (+)	*p* = 0.456	*p* = 0.501
	Nest protection	F = 19.225,	F = 10.565,	F = 0.351,
	(r^2^ = 0.033)	*p* < 0.001 **	*p* = 0.001 **	*p* = 0.554
	Regulations	F = 2.109,	F = 31.992,	F = 0.456,
	(r^2^ = 0.121)	*p* = 0.147	*p* < 0.001 **	*p* = 0.500
	Exclusions	F = 13.251,	F = 97.852,	F = 4.741,
	(r^2^ = 0.179)	*p* < 0.001 ** (+)	*p* < 0.001 ** (+)	*p* = 0.030 **
Convenience	On-ground protection	N/A	N/A	F = 13.355,
	(r^2^=N/A)			*p* < 0.001 **
	Exclusion and regulations	N/A	N/A	F = 9.924,
	(r^2^=N/A)			*p* = 0.002 **
Support for conservation	Ecosystem benefits	F = 7.409,	F = 0.113,	F = 0.533,
	(r^2^ = 0.018)	*p* = 0.007 ** (+)	*p* = 0.736	*p* = 0.466
	Single species benefits	F = 9.506,	F = 0.234,	F = 2.686,
	(r^2^ = 0.029)	*p* = 0.002 ** (−)	*p* = 0.629	*p* = 0.102

The degree of inconvenience reported in relation to on-ground managements and regulations and exclusions was negatively related to their perceptions of threats (Table 6). The more inconvenienced respondents were, the less effective they rated education/awareness, regulations and exclusions and the less supportive they were of plover conservation (Table 6). 

### 3.6. Frequency of Beach Use and Pet Ownership and Perceptions of Threats and Management

Similar proportions of dog walkers (94.5%) and non-dog walkers (93.6%) knew of the Hooded Plover. Awareness of a difference between the Hooded Plover and Masked Lapwing was 74.8% among non-dog walkers and 68.5% among dog walkers.

Frequency of beach use and dog walking did not influence the perceived effectiveness of different conservation managements. However, frequency of beach use influenced the level of inconvenience reported in relation to conservation management (Table 7). People who used the beach yearly were less inconvenienced by conservation management than those using the beach at weekly rates. Furthermore, respondents who walked their dogs on the beach reported more personal inconvenience in relation to exclusions and regulations than respondents without dogs (Table 7; 3.91 ± 0.08 *vs.* 4.41 ± 0.04, respectively). 

**Table 7:** Repeated measures ANOVA output on mean PCA scores for each identified reliable factor. ‘Dog walk’ referring to whether respondents walked their dog on beaches (0 = No, 1 = Yes) and frequency of beach use by respondents (where 1 = once a year to several times a year, 2 = several times a month to monthly, and 3 = daily to weekly), plus interaction term. Significant results are denoted as ** = *p* < 0.05.

Question	Frequence of use	Dog walk	Frequency of use x
	(df = 2)	(df = 1)	Dog walk (df = 2)
Threats	F = 7.243, *p* = 0.001 **	F = 0.112, *p* = 0.738	F = 4.175, *p* = 0.016 **
Effectiveness actions	F = 0.912, *p* = 0.403	F = 0.324, *p* = 0.569	F = 0.372, *p* = 0.689
Convenience	F = 4.577. *p* = 0.011 **	F = 13.225, *p* < 0.001 **	F = 2.049, *p* = 0.130
Support for conservation	F = 1.938, *p* = 0.145	F = 0.479, *p* = 0.489	F = 3.560, *p* = 0.029 **

**Figure 2:**
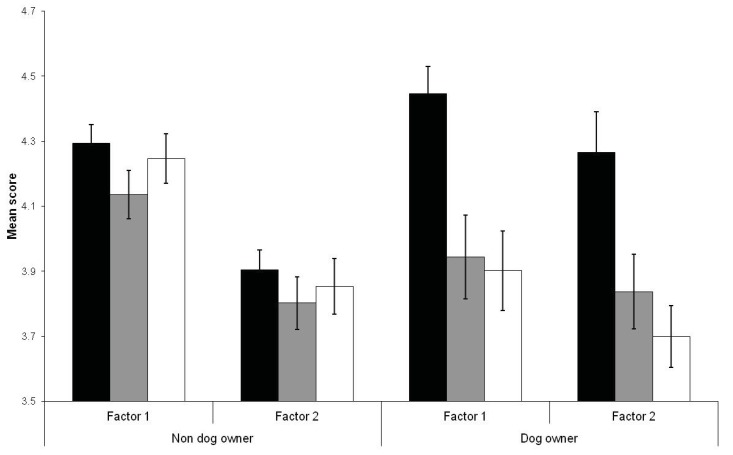
Mean scores (± one standard error) for factors for human-related impacts (Factor 1) and integrity of habitat (Factor 2) in relation to respondents beach use (black bars represent yearly, grey bars monthly, and white bars weekly) and dog walking on beaches (dog owners, non-dog owners).

**Figure 3:**
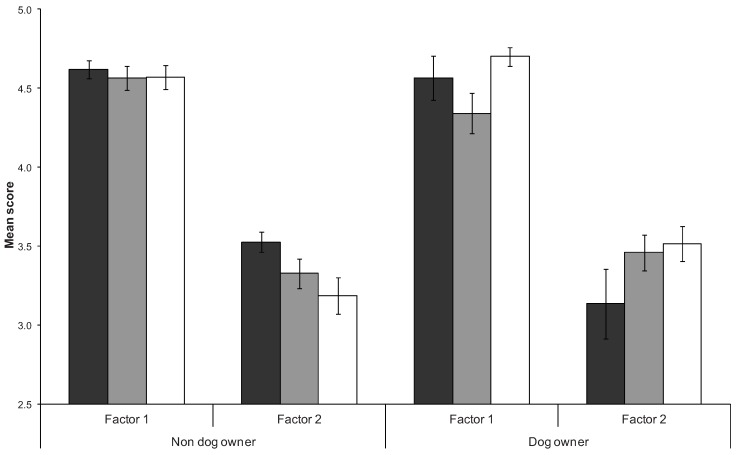
Mean scores (± one standard error) for factors related to ecosystem benefits of conservation (Factor 1) and single species benefits of conservation (Factor 2) in relation to respondents beach use (black bars represent yearly, grey bars monthly, and white bars weekly) and dog walking on beaches (dog owners, non-dog owners).

The interaction between frequency of beach use and dog walkers significantly affected how respondents rated threats to Hooded Plovers (Table 7). Non-dog walkers were more similar in their perceptions of threats regardless of how frequently they used beaches whereas dog walkers who used the beach least frequently rated threats significantly higher than those who used the beach at monthly and weekly rates (Figure 2). Furthermore, dog walkers who used the beach most frequently differed significantly in agreement towards conservation statements regarding Hooded Plovers (Table 7). Dog walkers who used the beach at a monthly rate were less supportive of ecosystem benefits than dog walkers using the beach at weekly rates, but this trend was not evident for non-dog walkers (Figure 3). The degree to which non-dog walkers were supportive of the ‘single species benefits’ factor was negatively related to their frequency of beach use, however, this relationship was reversed for dog-walkers (Figure 3). Mean factor scores were still strongly supportive of conservation statements despite trends for differences amongst beach users associated with their frequency of use and dog walking on beaches.

## 4. Discussion

Urbanisation increases the interaction between humans and wildlife, and these interactions can detrimentally influence wildlife [38,6]. However, few studies focus on the human dimension of these interactions. Specifically, few studies document stakeholder attitudes to wildlife and wildlife management, and more species-specific studies are needed [39,40]. Beach users in this study and on Florida and New Zealand beaches [11,30,41] were generally supportive of bird conservation and of beach-nesting birds. People are often predisposed to helping threatened species [42]. In additional to personal, socioeconomic and professional factors [43,44], species attributes also influence the way they are perceived by people. Perceptions of individual species typically derive from the influence of factors such as the phylogenetic relationship of the animal to people, the animal's presumed aesthetic value, its size, assumed intelligence, cultural and historic relationship, perceived dangerousness, likelihood of inflicting property damage, morphology, and mode of locomotion [24,41,45]. The Hooded Plover is benign to humans (though Masked Lapwings swoop [46]), and have a high aesthetic appeal when used as a flagship species [38], but is small and camouflaged, so often goes undetected by beach visitors. Despite general support, we describe influences on the perceptions of Hooded Plover management, namely inconvenience, awareness, frequency of beach use and pet ownership.

### 4.1. Inconvenience

Inconvenience refers to either an increased difficulty in, or prevention of, attaining a goal. General levels of inconvenience with management were low, as has been reported from Northland, New Zealand [30]. The plover managements deployed are specifically designed to maximise coexistence (minimise inconvenience). For example, humans are only excluded from small parts of Victorian beaches [8], whereas in other countries substantial or entire beaches are closed to the public [47]. The emphasis on management that promotes coexistence appears to have minimised inconvenience, which is expected to enhance compliance [22]; high compliance with Hooded Plover managements is evident on many Victorian beaches [9].

Inconvenience varied between the stakeholder groups we describe, conforming to our predictions, namely that higher inconvenience occurred among those with lower awareness, those using beaches more frequently and dog walkers. People with lower awareness may not have experienced management on beaches and therefore may have responded on the basis of preconceived ideas rather than personal experience [48]. Alternatively, being less familiar with the species and its plight, they may regard management that required any behavioural adjustment as being inconvenient. The result in relation to greater frequency of use being associated with greater inconvenience conforms to our prediction, and suggests that more frequent behavioural change is considered as more inconvenient. In Northland, New Zealand, more frequent beach visitors were more cautious regarding bird management approaches which limited beach usage [30]. Many respondents in this study who used the beach frequently may have developed traditions (norms) that pre-dated plover management. Indeed, awareness and cultural norms are key factors explaining the occurrence of pro-environmental behaviour [48]. This suggests that specialised education or support programs could target more frequent or less aware beach users. 

Dog walkers are required to make a greater behavioural change (*i.e.*, to leash their dog) in comparison with non-dog walkers, and many dog walkers do not leash their dogs when required to, suggesting resistance to the idea of leashing among at least a selection of dog walkers [33]. For dog-walkers, managements may prevent attainment of their goal (e.g., off-leash dog exercise) while non-dog walkers will still attain their goal (e.g., exercise or spending time on the beach). Additionally, there may be a degree of dissonance, whereby dog walkers unconsciously seek consistency in their beliefs and mental frameworks, by justifying their behaviour (lack of compliance with leashing laws) according to their own perception of their behaviour as low impact. Indeed, Williams *et al.* [33] found that dog walkers consistently viewed their own dog as having little or no impact on the birds but other people’s dogs as highly threatening to the birds’ welfare. This internal dissonance is consistent with the finding that dog walkers who frequently use the beach have high levels of support for plover conservation: sympathy and support seem to be present, but inconvenience acts as a barrier to compliance. 

### 4.2. Awareness

Awareness of Hooded Plovers was high amongst the sample population, although low levels of confusion persisted regarding differences between plover (threatened) and lapwing (superabundant) species, which potentially degrades levels of concern over the threatened Hooded Plover. High awareness (>80% of beach users) of birds of conservation significance and their protective measures on beaches was also evident from beaches in Northland, New Zealand [30]. Less frequent beach users were more likely to disregard signage regarding actively managed beach birds [30], perhaps because of lower awareness. 

People were generally aware of the threat they pose to Hooded Plovers and rated human threats more highly than others, including loss of habitat. However, awareness of threats was positively related to prior awareness suggesting education is effective at conveying ‘ownership’ of the problem [11]. Almost two decades of education regarding Hooded Plovers and their plight may have underpinned the high levels of awareness of the birds and the threats they face [49]. Interestingly, respondents rated education/awareness raising strategies as the least effective tools for conserving the Hooded Plover. This suggests that participants are unaware of the impact that education and exposure to this issue via local media, signage, brochures and contact with local volunteers on beaches has had on shaping their awareness. An association has been suggested between low awareness among recreationists of their negative influence on wildlife, and their support for management efforts [21,50]; this study reports both high awareness of the negative effects of certain recreation, and high support for management of those threats. 

### 4.3. Regularity of Beach Use and Pet Ownership

Australians tend to use their ‘local’ beaches more regularly than ‘non-local’ beaches, and so regularity of use may reflect different populations of recreationists [4]. Regular beach users, particularly those who are likely to be having an impact on Hooded Plovers (e.g., off-leash dog walkers [18]) tend to rate threats less severely than other beach users, perhaps because their behaviour is inconsistent with the belief that that they are causing harm to plovers, or perhaps because they have witnessed dog-plover interactions and consider the impacts negligible because they do not observe direct, obvious, physical consequences but rather a response which they consider benign [22,48]. Recreationists at least sometimes perceive they cause less stress to wildlife than they actually cause [50]. One feature of Hooded Plover conservation is that the adults persist in locations (even though their breeding may not be viable), so the presence of the birds may reinforce the idea that threats are overstated (“These birds have been here for as long as I have, they’re not threatened”). Such subtle consequences during human-bird interactions might underpin less pro-environmental behaviour [50]. 

### 4.4. Management Recommendations

This study has revealed the importance of limiting the perception of inconvenience via targeted awareness-raising and education, and by ensuring that on-ground managements optimise coexistence (*i.e.*, have taken into account the breadth of beach users and their desire for continued access to beaches). Where coexistence is deemed as ineffective at mitigating impacts to threatened species, then limiting or prohibiting access should be associated with the provision of alternative areas or times of use at alternative sites. Given the multi-jurisdictional and multi-tenure nature of beach management in most countries, this will require collaboration and cooperation between agencies in formulating balanced coastal access and zoning across the landscape. Involvement of communities in the decision-making via opportunities for public comment and information sessions will improve the efficacy of policy change [38].

Public acceptance of on-ground managements and policy changes is challenging for regular, local beach users, as for dog walkers, who have pre-established beach use norms (this study). Here, implementation should be gradual and coupled with education, in particular delivered by rangers during patrols (most participants in this study were more open to this delivery mechanism). Websites, social media, permanent signage and brochures were viewed as less effective, however, participants did prefer signage on the beach itself around the nest site and compliance with this management is very high [9]. Rimmer *et al.* [51] revealed that personalising the bird and creating an emotional connection between the dog walker and bird through signage content were important in delivering conservation messages to this particular audience.

## 5. Conclusions

Urbanisation in Australia is biased towards the coast, and results in direct changes to coastlines and increases the human usage of beaches. Wherever people and wildlife interact, different stakeholder groups span the spectrum from “support” to “opposition”, or from “coexistence” to “conflict”. Determining which factors place a person along this spectrum is a critical step in approaching behaviour change. Here we have identified low awareness and high inconvenience as correlates of attitudes toward threatened species conservation and their management. Understanding perceptions of management strategies could enhance community engagement, avoid unnecessary conflicts, and assist in fine-tuning managements to promote coexistence.
